# Correspondence

**Published:** 1907-10

**Authors:** Walter Wyman


### CORRESPONDENCE

THE SANITARY MILK PROBLEM—THE SPREAD OF DISEASE BY MILK—THE CO-OPERATION IN THE STUDY OF THIS QUESTION IS SOUGHT—RETURNS MUST BE MADE NOT LATER THAN OCTOBER 15, 1907.

*Editor Buffalo Medical Journal:*

SIR:—The following circular letter has been issued to state and local health officers and other sanitarians:

In the study of the sanitary milk problem undertaken by this bureau at the direction of the Secretary of the Treasury and the President, it is desired to make a compilation of all authentic cases in which disease has been spread by milk. This will include cases where milk has been the undoubted means of carrying an infectious disease to one or more persons. Whereas, in the light of present knowledge, the greatest interest centers in cases of typhoid fever, diphtheria, and scarlet fever spread by this means, yet the report of other diseases carried in this way is also desired.

It is believed that although many epidemics caused by milk have been reported in the printed reports of boards of health and in the medical journals, a greater number known to medical men have not been so reported.

If you will coöperate by reporting to this bureau upon the furnished form, or otherwise, any cases of disease or epidemics spread by milk of which you have knowledge, it will be greatly appreciated. An addressed envelope, which will require no postage, is sent for the return of any report that may be made. Reports, to be of service, should be returned not later than October 15, 1907.

Treasury department, bureau of public health and marine-hospital service, Washington, August 28, 1807.

WALTER WYMAN,
*Surgeon-General.*

---

NOMENCLATURE OF PROPRIETARY REMEDIES.—*Critic and Guide,* January, 1907, discusses this question and refers, as illustrative example, to sextonol. The word saves the trouble of writing "tabellæ e sodii, calcii, ferri, mangani, quininæ et strychninæ glycerophosphatibus" with the quantities. An attempt was made to get a druggist to prepare such tablets, but they deliquesced on the following day. An investigation disclosed that the apothecary had only the glycerophosphates of lime and soda in stock; he therefore left out the iron and manganese and used the sulphates of strychnine and quinine instead of the glycerophosphates. Besides, the prescription cost much more than the sextonal tablets do.

